# Case design and flow resistance in high-alpine caddisfly larvae (Insecta, Trichoptera)

**DOI:** 10.1007/s10750-022-04981-y

**Published:** 2022-10-14

**Authors:** Johann Waringer, Simon Vitecek, Jan Martini, Carina Zittra, Ariane Vieira, Hendrik C. Kuhlmann

**Affiliations:** 1 Division Limnology, Department of Functional and Evolutionary Ecology, University of Vienna, Djerassiplatz 1, 1030 Vienna, Austria; 2 WasserCluster Lunz, Dr. Carl Kupelwieser Promenade 5, 3293 Lunz am See, Austria; 3 Institute of Hydrobiology and Aquatic Ecosystem Management, University of Natural Resources and Life Sciences Vienna, Gregor-Mendel-Straße 33, 1180 Vienna, Austria; 4 Department of Ecology, University of Innsbruck, Technikerstrasse 25, 6020 Innsbruck, Austria; 5 Institute of Fluid Mechanics and Heat Transfer, TU Wien, Tower BA/E322, Getreidemarkt 9, 1060 Vienna, Austria

**Keywords:** Hydraulic stress, Drag coefficients, Drag reduction strategies, Trichoptera larvae

## Abstract

For evaluating hydraulic stress reduction strategies of caddisfly larvae, our study has three goals. First, creating a database on Reynolds numbers (*Re*) and drag coefficients valid for Limnephilidae larvae with cylindrical mineral cases. Second, evaluating the effects of submerged weight and biometry in cases with comparable length/width ratios. And third, collecting field data in an alpine environment for gaining insights into the hydraulic niches occupied by thirteen Drusinae species. Biometric data were subsequently combined with published Reynolds numbers and mean flow velocity data measured immediately upstream of Limnephilidae larvae at the moment of dislodgement. This provides drag coefficients for the range of Reynolds numbers obtained in the field. Data reveal that heavy cases strongly benefit from compensating drag by submerged weight, thereby enabling species to utilize high velocity spots, an important benefit for filtering species.

**Supplementary Information:**

The online version contains supplementary material available at 10.1007/s10750-022-04981-y.

## Introduction

Each roughness element at the bottom of a stream modifies its surrounding hydraulic field and is, in turn, subject to hydraulic stress exerted by the flow (Dingman, 1984). This applies to both sediment particles and macrozoobenthic organisms, such as lotic insect larvae. In the latter, hydraulic stress is mostly compensated by active efforts of the larvae to cling to the substrate and to submerged body weight, which can be largely increased by shells and cases constructed by the larvae. In the present study, we focus on the ecological implications of caddisfly cases as means of compensating hydraulic stress. Such an approach involves experiments and field measurements based on reliable descriptors of case shape and performance, some simple ones such as geometry, weight, volume, and length–width ratios, and some complex ones, such as drag and lift coefficients. Such an extensive database is the prerequisite to address the structural diversity observed in Trichoptera.

In fact, there is an amazing variety in caddisfly cases (Wiggins, 1996; Wallace et al., 2003; Holzenthal et al., 2007; Rinne & Wiberg-Larsen, 2017; Morse et al., 2019): cross-sections range from triangular (e.g., *Anabolia brevipennis* (Curtis, 1834)) over quadrangular (e.g., *Brachycentrus montanus* Klapálek, 1892) to circular (e.g., *Beraea pullata* (Curtis, 1834)). There are also turtle- and purse-shaped cases (e.g., *Agapetus fuscipes* Curtis, 1834; *Hydroptila occulta* Eaton, 1873), and construction materials cover a wide spectrum from mineral particles of various grain sizes over mollusc and testacean shells to woody debris, algal filaments, and plant and freshwater sponge fragments.

In lotic habitats, a tubular case constructed of mineral particles is the most abundant type (Waringer & Graf, 2011). Even so, tubular cases are very different in length, width, weight, curvature, and roughness. Generally they are always tapered, because after larval molts the larger instars add new case sections at the anterior end. This particular feature adds a certain degree of streamlining to the overall tubular case design. Moreover, as the legs of the larvae which hold on to the substrate extend from the anterior case opening, the longitudinal axis of the case aligns parallel with the flow (Waringer et al., 2021).

From a fluid dynamical perspective, such cases may be abstracted as near-wall, submerged, cylinder-like bodies exposed to a fluid flow where the maximum (anterior) case diameter defines how far the larva extends into the log-law layer in which turbulent fluctuations become important (Hoerner, 1965; Kuhlmann, 2014; Waringer et al., 2020). For the biota in question, the hydraulic stress on a body exposed to a flow is a force per area of the body which results from the fluid dynamical pressure (normal stress) and from viscous effects (normal and tangential [shear] stresses) (Scott, 2005). Unfortunately, basic biometric and hydraulic data sets necessary for estimating hydraulic stress parameters applicable to field conditions are lacking. The aim of the present study is, therefore, (1) to offer information on drag coefficients valid for the typical range of Reynolds numbers encountered by lotic caddisfly larvae, (2) to provide data on the type and amount of hydraulic stress for both viscous and pressure drag dominance, and (3) to detect and evaluate strategies of hydraulic stress reduction, based on case biometry and on series of in situ velocity measurements in the immediate, harsh, high-alpine, hydraulic environment of Drusinae larvae.

## Materials and methods

### Biometric parameters

A database on biometric parameters (case length, case width, anterior case surface) is necessary for the computation of hydraulic parameters such as Reynolds number and drag which describe case performance in the flow; weight and volume data are necessary for obtaining density of larvae and cases. In total, there are five larval instars in Limnephilidae, with mineral, cylindrical cases becoming longer and wider with each subsequent larval molt; for the biometrical database, we took larvae sampled in their alpine habitats and preserved in 70% Ethanol. Fresh weight of 72 final instar larvae in their cases belonging to thirteen Drusinae species were measured on a laboratory balance (to the nearest mg). For obtaining volume data of larvae plus cases (to the nearest 0.01 ml) a burette filled with water was used. For each individual specimen we removed excess conservative fluid with filter paper and took the difference between volume readings without and with larvae. Aspect ratios Γ were calculated by dividing mean case length *L* by anterior case width *2R*. Length and width data (to the nearest 0.01 mm) were measured under a dissecting microscope using an ocular micrometer. Calculations of the projected frontal surface areas were based on maximum (anterior) case diameters. For submerged weight reduced by buoyancy, the volume measurements of larvae plus cases were multiplied by the density of water at 10 °C and subsequently subtracted from fresh weight. The corresponding biometric data for the five larval instars of *Allogamus auricollis* (Pictet 1834) and fifth instars of *Potamophylax cingulatus* Stephens 1837 as well as velocity data acting exactly at the moment of dislodgement (drift entry) were taken from Waringer (1989, 1993) and supplemented with additional unpublished measurements. The biometric database is included in Tables 1 and 2.

**Table 1:** Case metrics and hydraulic parameters for the five larval instars of *Allogamus auricollis* (Pictet, 1834) (*A.a.* 1–*A.a.* 5) and for fifth instars of *Potamophylax cingulatus* Stephens, 1837 (*P.c.* 5) at the moment of dislodgement (drift entry) of dead larvae (in cases), combining data given by Waringer (1989) with unpublished data

Parameter	*A.a.* 1	*A.a.* 2	*A.a.* 3	*A.a.* 4	*A.a.* 5	*P.c.* 5
*L* (*x̅* ± 95% CL; mm)	3.21 ± 0.20	5.97 ± 0.65	10.32 ± 1.02	13.62 ± 0.86	16.82 ± 0.51	21.80 ± 2.74
Anterior case width (*x̅* ± 95% CL; mm)	0.95 ± 0.06	1.73 ± 0.13	2.47 ± 0.23	3.40 ± 0.23	4.70 ± 0.20	6.33 ± 0.42
Posterior case width (*x̅* ± 95% CL; mm)	0.59 ± 0.04	1.55 ± 0.19	2.35 ± 0.15	1.74 ± 0.01	3.40 ± 0.32	5.47 ± 0.42
Mean case width (*x̅* ± 95% CL; mm)	0.77 ± 0.05	1.64 ± 0.16	2.41 ± 0.19	2.57 ± 0.12	4.05 ± 0.17	5.90 ± 1.74
*F*_F_ for dead larvae (*x̅* ± 95% CL; × 10^–6^ N)	1.48 ± 0.33	4.13 ± 0.68	20.23 ± 4.26	36.55 ± 3.32	279.22 ± 36.22	1722.01 ± 27.0
*F*_F_ for living larvae (*x̅* ± 95% CL; × 10^–6^ N)	28.39 ± 4.97	65.90 ± 9.08	77.58 ± 9.30	134.10 ± 30.93	492.80 ± 38.09	no data
Ratio (*F* for dead/*F* for living larvae)	19.2	16.0	3.8	3.7	1.8	no data
U_*_ (*x̅* ± 95% CL; m s^−1^)	0.030 ± 0.001	0.049 ± 0.001	0.099 ± 0.016	0.123 + 0.008	0.209 ± 0.016	0.705 ± 6.01
Re* (*x̅* ± 95% CL)	84 ± 8	254 ± 32	888 ± 87	1457 ± 181	3057 ± 320	13,364 ± 2790

For calculations, a friction coefficient of 0.69 (Waringer, 1989) was taken

**Table 2:** Basic biometry of selected lotic Limnephilidae larvae showing instar, fresh and submerged weight, the percentage of submerged weight with respect to fresh weight, volume, projected frontal surface (± 95% CL), and aspect ratio

Species	Instar	Fresh weight (larva + case; mg)	Volume (larva + case; mm^3^)	Submerged weight (larva + case; mg); % fresh weight	Projected frontal surface (mm^2^)	Aspect ratio (l /w)
*Allogamus auricollis* (Pictet, 1834)	1	0.89 ± 0.19	0.67 ± 0.15	0.22 ± 0.05; 24.72	0.77 ± 0.05	3.38
*Allogamus auricollis* (Pictet, 1834)	2	3.68 ± 0.60	3.08 ± 0.54	0.61 ± 0.10; 16.58	2.96 ± 0.44	3.45
*Allogamus auricollis* (Pictet, 1834)	3	21.99 ± 4.60	19.03 ± 4.36	2.99 ± 0.63; 13.60	5.98 ± 1.02	4.18
*Allogamus auricollis* (Pictet, 1834)	4	30.36 ± 2.65	25.00 ± 3.62	5.40 ± 0.49; 17.80	9.45 ± 1.35	4.01
*Allogamus auricollis* (Pictet, 1834)	5	181.00 ± 23.65	140.00 ± 12.78	41.25 + 5.35; 22.79	17.36 ± 1.51	3.58
*Drusus alpinus* Meyer-Dür, 1875	5	44.00 ± 12.42	38.00 ± 16.19	6.07 ± 5.83; 13.80	6.01 ± 0.89	3.61
*Drusus franzi* Schmid, 1955	5	78.50 ± 19.34	60.00 ± 25.98	10.09 ± 0.05; 12.85	9.00 ± 2.16	3.80
*Drusus biguttatus* (Pictet, 1834)	5	47.64 ± 6.33	42.73 ± 11.67	8.46 ± 5.43; 17.76	7.92 ± 1.28	3.38
*Drusus chauvinianus* (Stein, 1874)	5	54.40 ± 23.65	40.00 ± 17.56	14.47 ± 13.77; 26.60	6.44 ± 0.75	3.70
*Drusus dudor* Oláh, 2017	5	52.00 ± 17.05	45.00 ± 19.63	10.47 ± 5.46; 20.14	7.76 ± 1.32	3.36
*Drusus flavipennis* (Pictet, 1834)/*Drusus rhaeticus* (Schmid, 1955)	5	18.00 ± 2.92	16.70 ± 2.01	3.73 ± 0.97; 20.72	3.50 ± 0.66	4.29
*Drusus mixtus* Pictet, 1834	5	69.14 ± 16.09	54.99 ± 11.10	14.25 ± 5.00; 20.61	8.01 ± 1.77	3.46
*Drusus monticola* McLachlan, 1876	5	88.33 ± 26.33	66.67 ± 26.67	21.79 ± 9.63; 24.87	9.65 ± 1.08	3.62
*Drusus chrysotus* (Rambur, 1842)	5	138.40 ± 50.57	102.00 ± 28.31	37.71 ± 32.05; 27.25	14.21 ± 2.76	3.24
*Drusus katagelastos* Vitecek, 2020	5	86.00 ± 2.48	66.67 ± 14.34	19.45 ± 12.23; 22.62	9.64 ± 1.37	3.76
*Drusus muelleri* McLachlan, 1868	5	110.00 ± 7.97	85.00 ± 9.19	25.15 ± 6.62; 22.86	13.89 ± 1.72	3.58
*Drusus nebulicola* (McLachlan, 1867)	5	19.67 ± 15.97	17.40 ± 11.02	4.03 ± 1.47; 20.49	4.01 ± 0.61	4.67
*Ecclisopteryx madida* McLachlan, 1867	5	87.00 ± 38.12	67.31 ± 35.31	19.81 ± 11.86; 22.77	8.07 ± 1.31	3.84
*Potamophylax cingulatus* Stephens, 1837	5	781.44 ± 20.00	527.50 ± 16.43	254.87 ± 7.62; 32.62	31.46 ± 4.01	3.44

### Hydraulic stress parameters

In streams and rivers, the unidirectional mean flow is driven by gravity and depends on the slope of the river bed. Lotic organisms on the stream bed are, therefore, subject to forces acting on the surface of their body (Morisawa, 1968; Vogel, 1981; Gordon et al., 1992). In the first step, we wanted to explore the character of those forces around Drusinae larvae in their cases by computing the relative importance of viscous and pressure forces expressed by the Reynolds number of the body.

1 $$\mathrm{Re}=\frac{L U}{\nu },$$

where *U* (m s^−1^) is the mean flow velocity to which the larva is exposed, *L* is a characteristic length of the larva (e.g., case length), and *ν* is the kinematic viscosity which is temperature dependent (lab: ν_15°C_ = 1.15 × 10^−6^ m^2^ s^−1^; field: ν_10°C_ = 1.30 × 10^−6^ m^2^ s^−1^). At very low Reynolds numbers, viscous forces (skin friction) on the body dominate. With increasing *Re*, pressure forces (form drag) become the dominant component of total drag.

In the second step, the magnitude of the hydraulic forces acting on the larvae was investigated. The force component parallel (streamwise) to the direction of the (mean) flow is called the drag, whereas the cross-stream component is the lift (Kuhlmann, 2014). Both are usually expressed by the non-dimensional drag and lift coefficients *C*_*D*_ and *C*_*L*_, which result when the drag and lift are scaled by the frontal stagnation pressure times a characteristic area *A*, which depends on body shape. For thick, blunt bodies, such as spheres, cylinders, and cars, the area projected in streamwise direction is typically selected, ‘that is, the area one sees when looking toward the body from upstream’ (White, 2011), which also applies for caddis larvae with cylindrical cases. For an approximation of the projected area, we take *A* = *πR*^*2*^, where *R* is the maximum radius of the case. For a blunt body, the total drag force *F*_D_ is usually expressed in terms of the stagnation point pressure times the cross-sectional area *A* which leads to

2 $${F}_{\mathrm{D}}= {C}_{\mathrm{D}} Ap = {C}_{\mathrm{D}}\frac{ A\rho {U}^{2}}{2}$$

where the drag coefficient *C*_D_ is introduced. *ρ* is the density of the water which is temperature dependent (lab: *ρ*_15°C_ = 999.13 kg m^−3^; field: *ρ*_10°C_ = 999.70 kg m^−3^). For moderately large Reynolds numbers (> 100) *C*_D_ is of the order of 1 with $A\rho {U}^{2}/2$ providing the order of magnitude of the drag force. The exact drag coefficient *C*_D_ depends on the shape of the body and the Reynolds number *Re* or, equivalently, on *U* (for constant *L* and *ν*). Typically, the drag coefficient is determined for a homogeneous flow (moving airfoil in quiescent air). But if the oncoming flow is turbulent the drag coefficient will change.

In the third step, we explored the stabilizing forces of a larva in its case, based on its submerged weight. For a body resting on the stream bed, the total drag force (2) is counteracted by a static friction force *F*_F_. Based on the submerged weight of the larvae in their cases this force can be estimated by

3 $$F_{{\text{F}}} = fVg(\rho _{L} - \rho ),$$

where *V* is the volume (larva + case; m^3^),* g* is the acceleration due to gravity (9.81 m s^−2^), *ρ*_L_ is the density of the larva including the case (kg m^−3^),* ρ* is the density of water, and *f* is an empirical friction factor which is* f* = 0.69 for mineral Trichoptera cases on mineral substrates (Waringer, 1989). In flattened body shapes, hydrodynamic lift may also be substantial. However, in benthic animals that are less or not at all dorsoventrally flattened, e.g., Plecoptera or Trichoptera larvae, the combined pressure and viscous skin friction drag by far outweigh the lift force, with the latter actually close to zero (Weissenberger et al., 1991).

As outlined above, the drag coefficient *C*_D_, necessary for calculating drag, will change if the oncoming flow is turbulent, which is frequently the case in the natural habitats of Drusinae larvae. In the fourth step, therefore, we had to provide information on *C*_D_ over the range of Reynolds numbers typical for lotic caddisfly habitats. For this, we used the particular situation in which *F*_D_ = *F*_F_. This is the dislodgement condition at the moment of drift entry

4 $$C_{{\text{D}}} \left( {Re^{*} } \right)A\frac{{\rho U^{{*2}} }}{2} = fVg(\rho _{{\text{L} - }} \rho ).$$

It occurs at a particular velocity U* or at a particular Reynolds number ${Re}^{*}$. Solving for the drag coefficient we obtain as follows:

5 $${{C}^{*}}_{\mathrm{D} }=\frac{2fg( {\rho }_{\mathrm{L}}- \rho )}{\rho } \frac{ V}{A {{U}^{*}}^{2}}$$

or with U* = ν Re^*^/*L*,

6 $$C_{{\text{D}}}^{*} = \frac{{2fg(\rho _{{\text{L}}} - \rho )}}{{\rho \nu ^{2} }}\frac{{VL^{2} }}{A}\frac{1}{{Re^{{*2}} }}.$$

For a cylinder-like body, we can assume *V* = *cAL* with constant *c* (*c* = 0.39) as shape parameter. The constant accounts for the fact that *V* is based on the anterior (maximum) case diameter which overestimates *V* for conical cases. Because we consider only the same universal shape, we can drop the dependence of *C*_D_ on *c* and obtain as follows:

7 $$C_{{\text{D}}}^{*} = \frac{{2cfg\left( {\rho _{{\text{L}}} - \rho } \right)}}{{\underbrace {{\rho \nu ^{2} }}_{K}}}\frac{{L^{3} }}{{Re^{{*2}} }} = K\frac{{L^{3} }}{{Re^{{*2}} }}.$$

In Eq. (7), the coefficient *K* is approximately constant over all taxa and instars, based on the fact that density variations were negligible in Drusinae larvae and cases. Therefore, the relation C*_D_ ~ *K* (*L*^*3*^/Re*^2^) captures the essential dependence of the drag coefficient at the moment of dislodgement on the size of the larva, parameterized by its length *L*. For this experimental determination of *C*_D*_, dead lotic Trichoptera larvae (preserved in 70% Ethanol) were used, because the dislodgement conditions expressed by Eq. (4) are only valid without active efforts of the larvae to cling to the substrate. We exposed the larvae in their cases to increasing flow velocities, using a shallow laboratory flume (described in detail by Waringer 1989a; water depth = 5–7 mm) and measured the dislodgement velocity U* at which they drifted from their initial position. Larvae were exactly aligned parallel to the incoming flow, heads upstream. Velocity at the moment of dislodgement was in the range of 0.03–0.75 m s^−1^ and measured immediately in front of the larvae.

In the step five of our analysis we deduced the expected drag as a function of the size of larvae and cases using a generalization of Eq. (7), where we need to know the mean velocity entering Re*. For this, Le Roux (2004) provided an integrated equation valid for the transitional flow regime between hydrodynamically smooth (= viscous sublayer thicker than roughness elements) and rough conditions (= viscous sublayer thinner than roughness elements):

8 $$\frac{U}{{{(gSy)}^{*}}^{0.5} }=2.5\mathrm{ln}\left(\frac{ Re y}{ k} \right)+5.3-0.1206 (Re-5)$$

with *Re* = $\frac{\rho k({{gSy)}^{*}}^{0.5}}{ \mu }$, where *S* is the slope (dimensionless), *y* is the water depth (m), *µ* is the dynamic viscosity (N s m^−2^), and *k* is the substrate roughness (*m*).

### Field measurements

Flow velocity data were collected in the field from 7 June to 25 July 2019, from 15 to 23 May 2021, and from 10 to 15 July 2021. The study sites were situated in high-gradient, low-order, unpolluted, shallow, and summer-cold mountain brooks 50 to 100 cm wide, with adjoining catchments consisting mostly of alpine grassland and, less frequently, mixed forest at some stations below the treeline. A synopsis of the sites is provided in Supplementary Material 1. In total, 101 data sets were taken for fifth instar larvae. The flow to which larvae are exposed in their natural setting is highly fluctuating (usually turbulent; Waringer et al., 2020) and even the mean velocity profiles (as functions of the distance from the ground) may vary. Furthermore, these flow details are very difficult to measure under field conditions. We were able, however, to measure the velocity ahead of the larvae by a tripod-mounted Schiltknecht MiniWater 20 Micro-velocimeter probe to the nearest 0.01 m s^−1^ (time resolution = 1 measurement per s). This probe has a circular cross section of 10 mm. The tripod enabled setting the measuring head, touching the bottom, directly at front center of a larva, at water depths ranging from 10 to 30 mm, thereby yielding a good approximation to the velocity field vertically averaged over the height of the larva. The mean velocity *U* is obtained as the arithmetic mean of the recorded fluctuating signal for each data set. Information on the number of single-velocity measurements per data set is included in Table 3.

**Table 3:** Flow velocity *U* (m s^−1^; mean with range), Reynolds numbers *Re* (mean with range), static friction (× 10^–6^ N; mean with 95% CL), and maximum drag (× 10^–6^ N) measured at the location of fifth instar Drusinae larvae in the field

Species	*U*	*Re*	*N*	Static friction (× 10^–6^ N)	Max drag (× 10^–6^ N)	Ratio
*Drusus alpinus* Meyer-Dür, 1875	0.14 (0.00–0.50)	1073 (0–3831)	361–2219	41.09 ± 39.46	295.01	7.2
*Drusus franzi* Schmid, 1955	0.05 (0.00–0.59)	494 (0–5832)	126–909	68.70 ± 0.34	448.59	6.5
*Drusus biguttatus* (Pictet, 1834)	0.35 (0.14–0.54)	2873 (1149–4432)	30–301	57.26 ± 36.75	406.43	7.1
*Drusus chauvinianus* (Stein, 1874)	0.20 (0.04–0.43)	1627 (0–3500)	30	97.95 ± 93.21	250.23	2.6
*Drusus dudor* Oláh, 2017	0.23 (0.00–0.36)	1861 (0–2913)	128–202	70.87 ± 36.96	242.60	3.4
*Drusus flavipennis* (Pictet, 1834)/*Drusus rhaeticus* (Schmid, 1955)	0.02 (0.00–0.11)	138 (0–762)	303–468	25.25 ± 6.57	27.98	1.1
*Drusus mixtus* Pictet, 1834	0.34 (0.25–0.41)	2874 (2114–3466)	570	96.46 ± 33.85	285.03	3.0
*Drusus monticola* McLachlan, 1876	0.21 (0.00–0.33)	2048 (0–3219)	70–435	147.49 ± 65.18	235.17	1.6
*Drusus chrysotus* (Rambur, 1842)	0.19 (0.00–0.54)	2008 (0–5707)	103–420	255.26 ± 216.96	603.05	2.4
*Drusus katagelastos* Vitecek, 2020	0.34 (0.00–0.93)	3445 (0–9422)	96–603	131.66 ± 96.33	832.58	6.3
*Drusus muelleri* McLachlan, 1868	0.25 (0.00–0.57)	2894 (0–6599)	302–439	170.24 ± 44.81	588.90	3.5
*Drusus nebulicola* (McLachlan, 1867)	0.56 (0.03–1.38)	4523 (242–11,146)	23–803	27.28 ± 9.95	672.14	24.6
*Ecclisopteryx madida* McLachlan, 1867	0.03 (0.00–0.15)	284 (0–1419)	303–731	134.09 ± 80.28	75.19	0.56

*N* = number of single-velocity measurements (time resolution = 1 measurement per s). Ratio = maximum drag experienced in the field/static friction defined by submerged weight. Data are based on a water density of 999.70 (10 °C)

## Results

### Biometry

The biometric database includes information for 16 taxa of Limnephilidae larvae (subfamilies Drusinae and Limnephilinae–Stenophylacini), most in the last (fifth) instar, but also for instars 1–4 in *Allogamus auricollis* (Pictet 1834). For the close species pair *Drusus rhaeticus* (Schmid 1955) and *Drusus flavipennis* (Pictet 1834), data were pooled. Based on weight and overall size of fully grown larvae, the largest taxon included was *Potamophylax cingulatus* Stephens 1837, whereas *D. rhaeticus/flavipennis* was the smallest. Taxa-specific data on case biometry necessary for the calculations of hydraulic parameters, such as static friction and Reynolds numbers corresponding to the drift entry velocity U^*^, are summarized in Tables 1 and 2.

### Drag coefficient

**Fig. 1:**
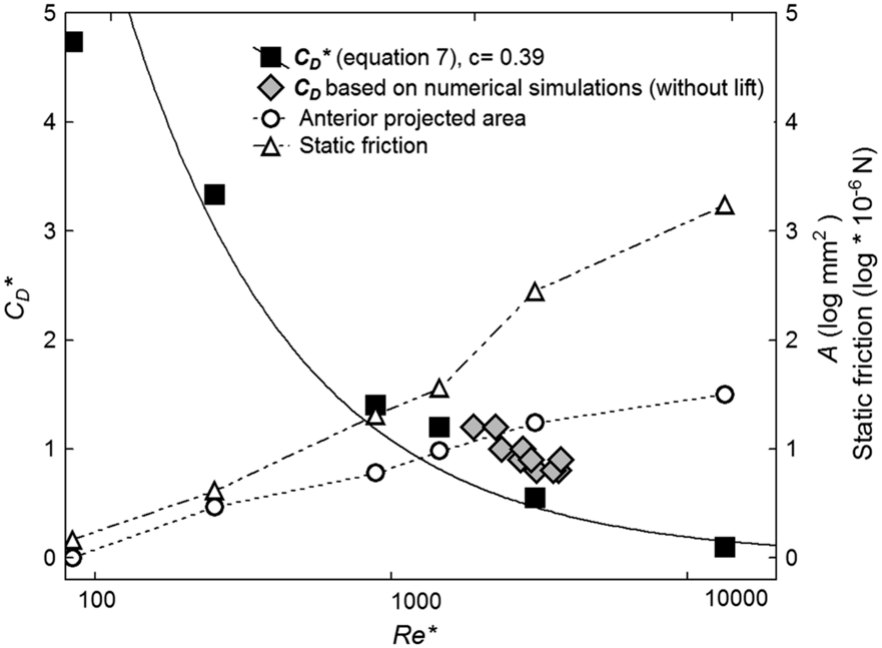
Projected frontal case area *A* (log mm^2^; circles), static friction (log × 10^–6^ N: triangles), and drag coefficient *C*_D_* (Eq. (7) with *c* = 0.39; squares) as a function of the Reynolds number Re* at the moment of dislodgement for first to fifth instar larvae of *Allogamus auricollis* (Pictet 1834) and fifth instar larvae of *Potamophylax cingulatus* (Stephens 1837). Solid regression line for data points of *C*_D_* according to equation: *C*D* = 193 * (Re*)^−^.^0.75^ (*P* < 0.000; *r*^2^ = 0.92). Data are based on unpublished and experimental flume data (Waringer 1989, 1993). Diamonds: data of numerical OpenFoam simulations conducted with surface models of *D. alpinus* Meyer-Dür, 1875, *D. bosnicus* Klapálek 1899, *D. discolor* (Rambur 1842), *D. monticola* McLachlan, 1876, and *D. nebulicola* (McLachlan 1867) by A. Vieira and H.C. Kuhlmann.

Drag coefficients *C*_D_* are calculated using Eq. (7) and are based on dislodging flow velocities ranging from 0.030 m s^−1^ in first instar *A. auricollis* larvae to 0.209 m s^−1^ in fifth instars when the longitudinal body axes were exactly aligned with flow, heads directed upstream. Due to their high submerged fresh weight of up to 255 mg, which is six times higher than in *A. auricollis* (fifth instars), drift entry of final instar larvae of *P. cingulatus* was measured at drift entry flow velocities (U*) of 0.71 m s^−1^ (Tables 1 and 2). Reynolds numbers at the moment of dislodgement (Re*) were below 100 in first instar larvae of *A. auricollis*, between 100 and 1000 in instars 2 and 3, and between 1400 and 3100 in instars 4 and 5; for final instar larvae of *P. cingulatus*, Re* was one order of magnitude higher than in *A. auricollis* (Re* = 13,364; Fig. 1, Table 1). This reflects the rapidly increasing static friction and lower anterior surface (*A*) increase of larvae during ontogenesis. Fresh weight and, therefore, static friction were low in first to fourth instars (0.89–30.36 mg and 1.48–36.55 × 10^–6^ N, respectively), but strongly increased in fifth instar larvae of *A. auricollis* and *P. cingulatus* (181.00–781.44 mg and 279.22–1722.01 × 10^–6^ N, respectively), thereby increasing U* and Re* accordingly.

The anterior projected area *A* increased from 0.77 to 17.36 mm^2^ in first to fifth instar larvae of *A. auricollis* and was as high as 31.46 mm^2^ in final instars of *P. cingulatus*, reflecting the enlargement of the cases at a ratio of 1.3 to 1.9 in length between two successive molts. As surface increases with the power of 2 of the length, but volume with the power of 3, static friction due to submerged weight increased more rapidly than *A* in Fig. 1.

### Influence of case size on drag

**Fig. 2:**
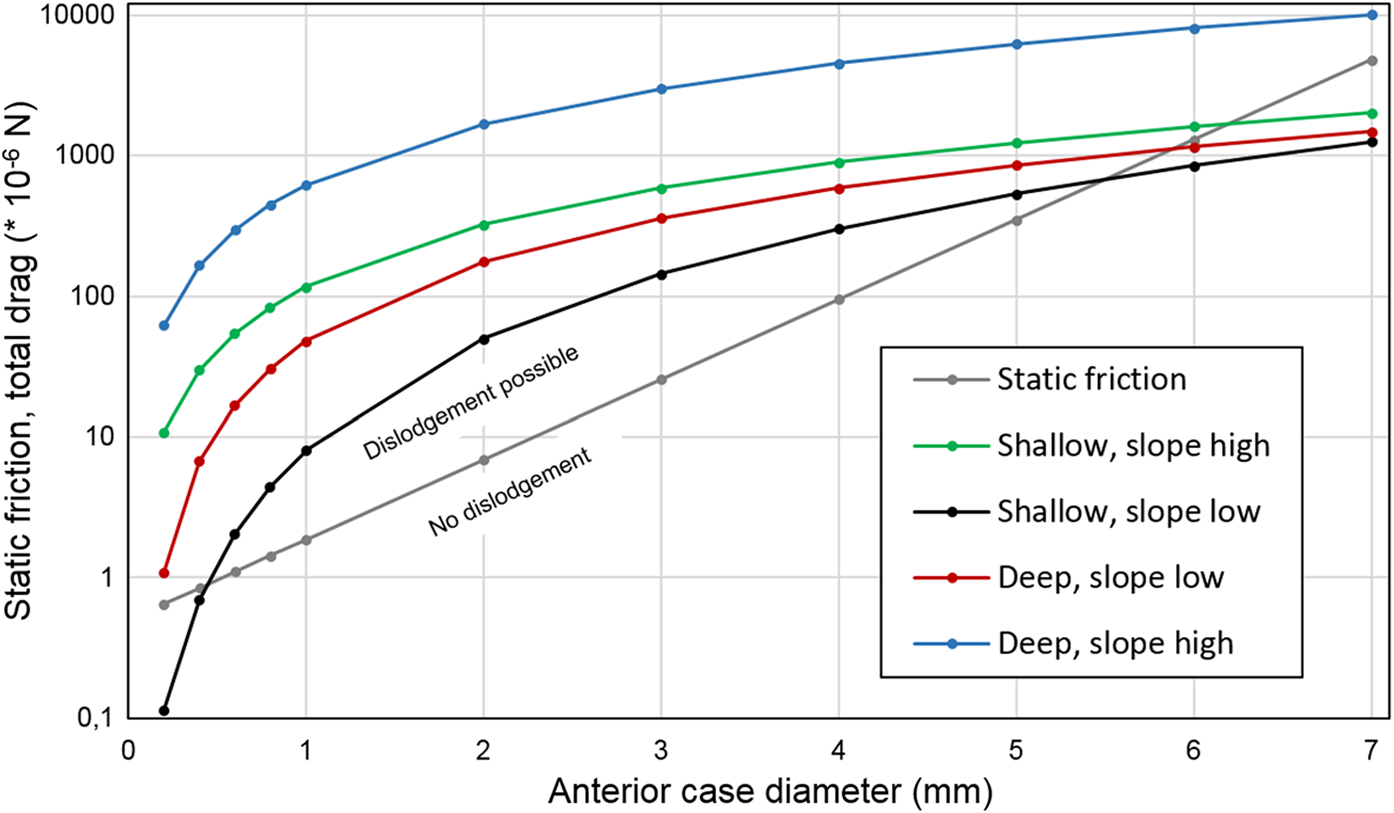
Log-linear relationship between maximum anterior diameter (mm) of cylindrical cases composed of mineral particles, static friction force *F*_*F*_ (gray line), and total drag force *F*_*D*_(lines in color) for four chosen situations: slope = 0.016 (‘slope high’) and 0.004 (‘slope low’); and mean water depth = 0.1 m (‘deep’) and 0.01 m (‘shallow’). Graphs are based on Eq. (8) and the following parameters: aspect ratios of cases = 3.5; water temperature = 10 °C; and mean density of larvae and cases = 1251.43 ± 47.09 kg m^−3^. Cases aligned with flow, larval heads directed upstream

In order to accommodate a given body volume, case sizes vary during ontogeny and between species (e.g., Table 1). Despite a high range of fresh weights (0.89–781.44 mg; Table 2), variation in aspect ratios (Γ; case length divided by anterior case width) was negligible, ranging from 3.24 to 4.67 (Table 2). To explore the interplay between hydraulic stress and submerged weight as well as maximum case height, we computed static friction *F*_F_ using Eq. (3) and total drag *F*_D_ using Eq. (2) for two water depths (0.1 and 0.01 m), two slope situations (0.004 and 0.016), and for case diameters in the range of 0.2 to 7 mm, using biometric data included in Tables 1 and 2. The drag force, shown in Fig. 2, is calculated using the fit for the drag coefficient. All evaluations are based on velocities valid for anterior case center (*R*) using Eq. (8) valid for the transitional flow regime between hydrodynamically smooth and rough conditions. In the species set investigated in the present study (Tables 1, 2), maximum anterior case diameters were always 2*R* ≥ 0.95 mm. Therefore, the drag gradients for case diameters 2*R* = 0.2–0.8 mm included in Fig. 2 are intended just to illustrate drag for putative small larvae.

Generally, the drag force for all case diameters is higher when the slope is high. The drag increases asymptotically with increasing case diameter (Fig. 2). This is due to the fact that small larvae benefit from their small anterior case diameters which experience less drag due to their smaller projected anterior surface *A* and low spot velocities near to the sediment surface. In the region below the interception from the gray static friction line and the total drag force curves, dislodgement of the larvae is not possible because total drag is lower than stabilizing static friction. This situation applies to hypothetical larvae with a case diameter of 2*R* = 0.2–0.4 mm in situation ‘shallow, slope low,’ and to case diameters 2*R* ~ 6 mm in all situations except ‘deep, slope high.’ In the regions above the interception from the friction line and the total drag curves, there is a need of active stabilizing efforts by the larvae in order to remain stationary.

### Drag experienced by Drusinae larvae in the field

**Fig. 3:**
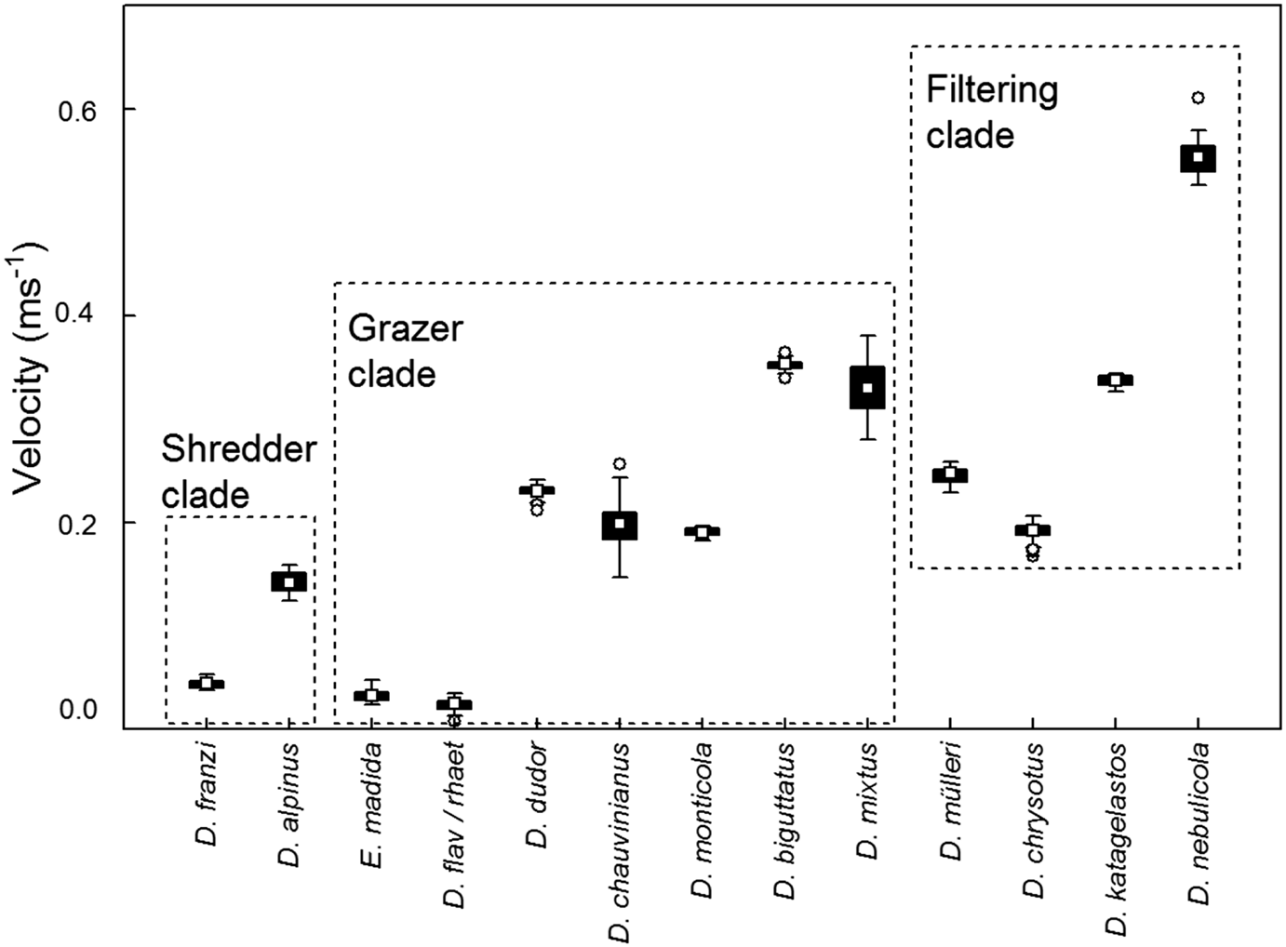
Flow velocities (m s^−1^) measured at front center of larvae of 13 Drusinae species in the field. The intervals between single measurements were 1 s, and the number of single-velocity measurements for each of the 101 data series is indicated in Table 3. White squares = means, black rectangles = 25/75% quartiles, error bars = range without outliers, circles = outliers

The 101 data sets taken at the locations of 13 Drusinae species at high-alpine sampling locations in Austria, Italy and Switzerland were used for exploring actual drag encountered by cased caddis larvae in the field. During all measurements, larvae were always aligned with flow, heads directed upstream. With respect to flow velocity, significant (*P* = 0.000; Kruskal–Wallis-ANOVA) differences were observed between feeding types as defined by Pauls et al. (2008) and Vitecek et al. (2015): in shredders which feed on roots, water mosses, riparian vegetation, and coarse particulate organic matter, mean velocities (± 95% CL) were as low as 0.09 ± 0.01 m s^−1^. In grazing species using epilithic biofilms as primary food source, we observed intermediate mean velocities of 0.19 ± 0.02 m s^−1^; highest mean velocities were recorded in carnivorous filter feeders (0.33 ± 0.03 m s^−1^) (Fig. 3). Whereas some time-resolved data series included several zero readings at low-stress larval locations, flow velocities in certain species were exceedingly high: in *Drusus katagelastos* up to 0.93 m s^−1^ and in *D. nebulicola* up to 1.38 m s^−1^. Corresponding Reynolds numbers ranged from 0 to 11,146 (Table 3). For shredders, mean *Re* (± 95% CL) was 756 ± 87, for grazers 1632 ± 136, and for filter feeders 3125 ± 162.

Calculated mean static friction using Eq. (3) and biometric information provided by Table 2 for the Drusinae species set studied in the field ranged from 25.25 × 10^–6^ N in the *Drusus flavipennis*/*rhaetica* (= *Metanoea*) species pair to 255.26 × 10^–6^ N in *D. chrysotus* (Table 3), indicative for the heavy cases and large larvae of the latter species. Mean drag exerted on the larvae was as low as 3.34 × 10^–6^ N in *D. flavipennis*/*rhaetica*, but up to 236.98 × 10^–6^ N in *D. katagelastos*; finally, maximum drag ranged from 27.98 × 10^–6^ N in *D. flavipennis*/*rhaetica* to 832.58 × 10^–6^ N in *D. katagelastos* (Table 3).

**Fig. 4:**
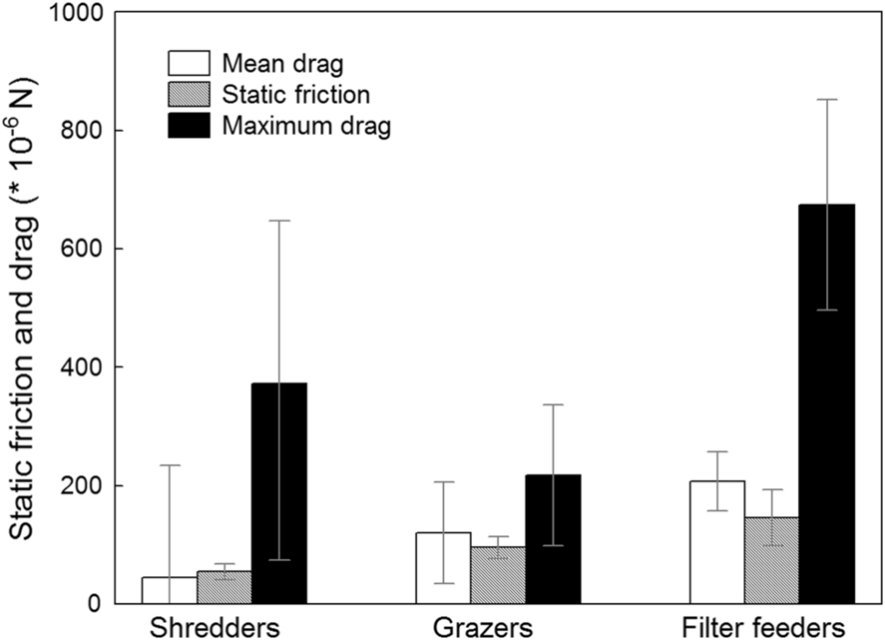
Static friction, mean, and maximum drag (means with SE; × 10^−6^ N) measured at the locations of 101 Drusinae larvae in high-alpine brooks. Data were pooled for the three feeding guilds of shredders, grazers, and carnivorous filter feeders

When grouped into feeding guilds established for Drusinae (Pauls et al., 2008; Vitecek et al., 2015), we noticed significant (*P* < 0.05) differences in mean and maximum drag forces, whereas differences in static friction were not significant (*P* > 0.05; Kruskal–Wallis-ANOVA). Static friction and mean drag increased from shredders over grazers to filtering collectors (Fig. 4). Interestingly, mean drag was smaller than static friction in shredders, indicating that *Drusus alpinus* and *D. franzi* frequented hydraulic spots where their submerged weight prevented drift entry and dislodgement. In grazers and filtering collectors, larvae have to cling actively to the substrate in order to stay stationary. Maximum drag in filtering collectors was as high as 674.17 × 10^–6^ N; in shredders, observed maximum drag was higher than in grazers (371.80 versus 217.52 × 10^–6^ N) (Fig. 4).

## Discussion

In their seminal paper on hydraulic stream ecology, Statzner et al. (1988) highlighted their observation that body shapes of many lotic zoobenthic organisms are not well adapted to minimize the hydrodynamic forces on their body. This also fully applies to the lotic caddisfly larvae of the present study: for example, in *Drusus chrysotus*, a deep concavity on the head capsule is present, and the pronota of Drusinae are frequently fitted with dorsal humps and ridges, thereby increasing the frontal area directed toward flow (Waringer & Graf, 2011). Despite those morphological drawbacks, the same species are abundant in stream sections where flow velocity and hydraulic stress are high simply because of nutritional and/or respirational needs (Waringer et al., 2021). In the present study we address this ecological trade-off by providing data on the hydraulic forces exerted on lotic caddisfly larvae and by highlighting avoidance strategies against hydraulic stress.

### Biometry

For the range of lotic Limnephilidae larvae measured (Table 2), the mean density for larvae plus cases, based on fresh weight, was found to be 1251.43 ± 47.09 kg m^−3^ which is significantly higher than the density of water at 10 °C (= 999.70 kg m^−3^), a water temperature frequently recorded in the field at the sampling sites of the species set. However, this stabilizing effect of higher density cannot be fully utilized by lotic caddisflies, because the proportion of submerged weight to fresh weight (mean ± 95% CL) was only (21.02 ± 2.30) %, illustrating the fact that submerged cased caddis larvae loose up to four-fifths of their fresh weight due to buoyancy which greatly reduces static friction, the most important stabilizing force in streams. Projected frontal surface areas (*A*) ranged from 0.77 ± 0.05 in first instars of *Allogamus auricollis* to 31.46 ± 4.01 mm^2^ in fifth instar larvae of *Potamophylax cingulatus*, almost 41 times as large as in the former taxon. As drag and lift are scaled by the frontal stagnation pressure times frontal surface area (*A*), this biometric parameter also strongly influences hydraulic stress experienced by the larvae: small cases generally experience less drag due to their smaller projected anterior surfaces.

### Drag coefficient

Knowledge of the range and magnitude of the drag coefficient *C*_*D*_ is a prerequisite for computing hydraulic forces acting on lotic caddisfly larvae. By taking case length as length parameter *L* in the equation of the organismic Reynolds number (Eq. 1), our laboratory flume experiments show that *C*_D_ =  >  > 1 for *Re* = 84 (Fig. 1), indicating the dominance of viscous (skin friction) drag*.* Experimental data for passive drift entry of first to fifth instar larvae of *A*. *auricollis* at Re* = 84 to 3057 resulted in *C*_D_* values decreasing from 4.3 to 0.73; in fifth instars of *P. cingulatus* at Re* = 13,364, *C*_D_* was as low as 0.22. Our numerical simulation data (unpublished) on fifth instar larvae of the 5 Drusinae species included in Fig. 1 (*Re* = 1902–3741, *C*_D_ = 1.29–0.84) were close to the established relationship of *C*_D_*(Re*). It must be kept in mind that Eq. (7) was derived for the condition of dislodgement only. It does not provide an expression for the drag as a function of the Reynolds number *C*_D_(*Re*). Only if the functional dependence *C*_D_(*Re*) for all larvae investigated would be the same, the data for the drag coefficient at the moment of dislodgement C*_D_ would sample the function *C*_D_(*Re*) at the sampling points Re*. However, as the numerical simulations performed independently fit the regression line from the dislodgement experiments quite well, the assumption C*_D_(Re*) = *C*_D_(*Re*) can be made. This means that *C*_D_ values for all species investigated are the same at a given *Re*, based on the uniformity of their mineral, conical cases, and the same aspect ratios. Therefore, the regression equation included in the legend of Fig. 1 can be taken to obtain *C*_D_ values for hydraulic parameter computations based on our data obtained in the field.

Of course, *C*_D_ and *C*_D_* always consist of a pressure and viscous drag component which could not be separated in the laboratory flume experiments. However, data generated by numerical simulations revealed that in *Drusus alpinus* Meyer-Dür, 1875 at *Re* = 3680 pressure drag contributed 43% and viscous drag 57% to total drag.

By taking a close look at the drag coefficients over the Re* range of 80 to 14,000 (Fig. 1), *C*_D_* strongly increases at Reynolds numbers smaller than 200, because viscous effects become more and more important. At *Re* 1000–2000, the *C*_D_* gradient becomes weaker. When looking at individual data points (black squares), a plateau effect becomes visible at *Re* 1000–2000, with a subsequent stronger decrease at *Re* > 2000 (Fig. 1). This pattern matches the *C*_D_(*Re*) gradient of a sphere in a flow field, where a plateau (or even minimum) of ~ 0.4 was also observed at intermediate *Re*. At low *Re*; however, *C*_D_ is much higher for the rougher and longer larvae than for spheres: at an increase of the aspect ratio Γ from 1 (sphere) to 4 (larvae), skin friction drag is also four times as high in the cylindrical cases than in spheres.

### Influence of case size on drag

In addition to the relationship *C*_D_ (*Re*) discussed above, our data summarized in Fig. 2 illustrate the effect of flow velocity on cases with the same length-to-width ratio. Generally, a smaller larva with a smaller anterior case diameter experiences less drag due to its smaller projected anterior surface *A* and because it is closer to the bottom where friction and the no-slip condition produces strongly declining spot velocities at the level of the case center (Fig. 2). As volume increases to the power of 3 of the length, static friction due to submerged weight strongly increases with the size of the larva and becomes dominant over total drag; this is shown in Fig. 2 where curve sections to the right of intersections between drag and static friction lines define situations where dislodgement is not possible. To the left of intersections, however, there is a need of active stabilizing efforts by the larvae in order to remain stationary. The data suggest that in intermediate-sized larvae the investment of muscle energy in their legs to remain stationary is highest and such size classes are more prone to dislodgement than small and very large larvae. This is well illustrated by *P. cingulatus* (fifth instars) where the volume gain (volume of larvae + cases divided by projected anterior area) is up to 16.77 mm^3^ mm^−2^ when compared with first instar larvae of *A. auricollis* (0.87 mm^3^ mm^−2^). Bulky cases benefit from compensating a large amount of drag: in *P. cingulatus*, a flow velocity of 0.71 ± 0.06 m s^−1^ is needed for drift entry of dead larvae in their cases aligned with flow (Waringer, 1993). Another strategy to remain stationary in the flow was observed in *Brachycentrus* spp.: in this caddisfly genus, larvae attach their cases to the substrate using silk to release their legs for filtering and other activities (Gallepp, 1977).

### Drag experienced by Drusinae larvae in the field

For our field measurements in the high-gradient, turbulent channels well above the treeline inhabited by the Drusinae larvae studied in detail, it is important to keep in mind that our considerations did not take into account the shape of the mean velocity profile of the turbulent flow. The velocity profile will enter the drag force and thus the drag coefficient such that *C*_D_ will no longer be unique. Furthermore, we did not account for any variation of the larva shape but assumed that all larvae had the same aspect ratios and are characterized by a common constant *c*. Finally, the mean velocity at the height of the larvae has only been approximated by our field measurements and are strictly valid only for 5-mm wall distance. Our data revealed that Drusinae species were able to deal with quite high flow velocities: *D. nebulicola* was able to withstand flow velocities of up to 1.38 m s^−1^. As all sampling locations were shallow, our flow velocity measurements are valid for the hydraulic situation at the larval positions. In *D. nebulicola*, the anterior case diameter is only 1.0–2.5 mm (Bohle, 1987); the latter, combined with a very high active effort of the larva in clinging to the substrate, resulted in withstanding a maximum drag of 672.14 × 10^–6^ N, equal to 25 times its static friction, illustrating the capacity of filter feeders to expose themselves to high flow velocities. The opposite trend was observed within the hydraulic environment of *D. flavipennis/rhaeticus* where the lowest flow velocity range (0–0.11 m s^−1^) and the lowest mean Reynolds number (*R*_*e*_ = 138) were observed. At such *Re* values, the species pair is situated in the descending left limb of the *C*_D_ graph. The highest submerged weight of 37.71 mg of the Drusinae species set was measured in *D. chrysotus*; although in this species the active effort of the larvae to cling to the substrate was one of the lowest of the species studied (only 2.4 times of submerged weight; Table 3), their high static friction and, hence, *Re* of up to 5707 shifted *C*_D_ values well into the right descending limb of Fig. 1.

At the level of feeding guilds, our new data are in line with the findings of Waringer et al. (2021). We noticed increasing trends in static friction and mean drag from shredders over grazers to filtering collectors (Fig. 4). Generally, mean drag was smaller than static friction in shredders, indicating their preferences for hydrodynamic low-stress patches within their predominantly low-stress (hypo-)krenal habitats where the accumulations of detrital particles, roots and riparian vegetation are highest. Grazers prefer hydrodynamic intermediate stress patches where the autotrophic biofilms and epilithic algae growing on mineral substrates are not yet eroded by flow velocities high enough to set sediments in motion. In line with the latter, maximum drag was lowest in this feeding guild (Fig. 4). Finally, species of the filtering clade frequent high stress patches because high flow velocity is required to operate their filtering apparatus efficiently, resulting in the highest observed maximum drag values in this group. Interestingly, in the filtering Drusinae clade, Zittra et al. (2022) detected modifications of the internal head capsule morphology such as an increasing simplification of the tentorium. In this regard, it can be assumed that the modified head capsules of the filtering carnivore Drusinae, e.g., dimples separated by fortified ridges, offer greater mechanical stability due to their structured surface as compared to the simply rounded head capsules of the other Drusinae, rendering the dorsal branch of the tentorium superfluous. This interpretation of the observed structures on the head capsule are doubtlessly capable of withstanding greater hydraulic forces, identifying the latter as important drivers for the evolution of morphological structures.

## Conclusion

Our study revealed that high-alpine caddisfly larvae cover a wide spectrum of hydraulic microhabitats in their steep and hard substrate channels. For the chosen set of thirteen species of *Drusus* and *Ecclisopteryx* species, our field measurements revealed Reynolds numbers ranging from 0 to 11,146, corresponding to instantaneous flow velocities at the larval locations from 0 to 1.38 m s^−1^. Drag coefficients calculated from experimental data in a laboratory flume (Waringer, 1989) yielded precise information on the drag acting on the larvae in the field. When grouping the chosen species set in functional feeding guilds, shredders frequented hydraulic spots where their submerged weight alone prevented drift entry and dislodgement, without the need for investing muscle energy in order to stay stationary. In grazers and filtering collectors, larvae have to actively cling to the substrate to prevent dislodgement. Grazer clade species expose themselves to only very low Reynolds numbers (e.g., *Drusus flavipennis/rhaeticus*). They achieve stress reduction by their small cases which experience less drag due to their smaller projected anterior surfaces and because close to the bottom, friction and the no-slip condition produce strongly declining spot velocities at the level of the case center. Another grazer strategy is taken by voluminous larvae constructing heavy, bulky cases such as *D. monticola* and *Ecclisopteryx madida*; they benefit from compensating a large amount of drag by submerged weight, thereby minimizing energy investments in active flow compensation. Filter feeders need to expose themselves to high hydraulic stress in order to efficiently operate their filtering apparatus.

## Supplementary Information

Below is the link to the electronic supplementary material.

Supplementary file1 (DOCX 14 kb)

## Data Availability

The data sets generated during the current study are available from the corresponding author on reasonable request.
