# Supplementary material for: Case design and flow resistance in high-alpine caddisfly larvae (Insecta, Trichoptera)
Source: Hydrobiologia. 2022 Oct 14;849(19):4259–71. doi: 10.1007/s10750-022-04981-y (PMC9613749; doi:10.1007/s10750-022-04981-y)
Supplement: Supplementary file 1 — Supplementary file1 (DOCX 14 kb) [file 10750_2022_4981_MOESM1_ESM.docx]

**Supplementary Material 1.**

Synopsis of sampling stations used in the present study.

Country Stream , Location Coordinates; elevation (m)

Austria Schwarze Sulm, Weinebene 46°50′ N, 15°01′ E; 1580

Mühl, Haslach 48°34′, 14°02′ E; 499

Italy Eggerbach, Antholz 46°52′ N, 12°06′ E; 1300

Oswaldbach, Merano 46°41′ N, 11°41′ E; 2280

Stream, Valchiusella 45°31′ N, 7°39′ E; 1560

Stream, Fondo 45°30’ N, 07°42′ E; 1584

Lago del Gias del Prete 45°31’ N, 07°38′ E; 2222

Switzerland Torrent d'Alléves, Grand Combin 45°58′ N, 7°14′; 2131

Torrent de Palasui, Grand Combin 45°59′ N, 7°13′ E; 2345

Torrent, Grand Combin 46°01′ N, 7°13′ E; 2234

Torrent, Grand Combin 45°51′N, 7°14′E; 1881

Torrent, Grand Combin 45°51′ N, 7°134′ E; 1912

Schwandbach, Obwalden 46°54′ N, 8°11′ E; 1379

Rio Foch, Ticiono 46°29' N, 8°45' E: 1000

**Appendix**

*List of symbols*

*A* Projected area (m²)

*C_D_* Drag coefficient (dimensionless)

*C_D_^*^* Drag coefficient at the moment of dislodgement (dimensionless)

*c* Shape parameter (dimensionless)

*F_D_* Total drag force (N)

*F_F_* Static friction force (N)

*f* Friction factor (diemensionless)

*g* Acceleration due to gravity (9.81 m s^-2^)

*K* Constant (m^-3^)

*k* Substrate roughness (m)

*L* Case length (m)

*M* Fresh weight (kg)

*p* Pressure (N m^-2^)

*R* Maximum radius of the case (m)

*Re* Reynolds number (dimensionless)

*Re** Reynolds number at the moment of dislodgement (dimensionless)

*S* Slope (dimensionless)

*U* Flow velocity (m s^-1^)

*U** Flow velocity at the moment of dislodgement (m s^-1^)

*V* Volume (m³)

*y* Water depth (m)

*Γ* Aspect ratio (dimensionless)

*µ* Dynamic viscosity (N s m^-2^)

*ν* Kinematic viscosity (m^2^ s^-1^)

*ρ* Density of water (kg m^-3^)

*ρ_L_* Density of the larva including the case (kg m^-3^)
